# Pulsed Radiofrequency of Lumbar Dorsal Root Ganglion for Chronic Postamputation Phantom Pain

**DOI:** 10.5812/kowsar.22287523.3768

**Published:** 2012-01-01

**Authors:** Farnad Imani, Helen Gharaei, Mehran Rezvani

**Affiliations:** 1 Department of Anesthesiology and Pain Medicine, Rasoul-Akram Medical Center, Tehran University of Medical Sciences (TUMS), Tehran, Iran

**Keywords:** Phantom Limb, Pulsed Radiofrequency Treatment, Spinal Ganglia

## Abstract

Chronic pain following lower-limb amputation is now a well-known neuropathic, chronic-pain syndrome that usually presents as a combination of phantom and stump pain. Controlling these types of neuropathic pain is always complicated and challenging. If pharmacotherapy does not control the patient’s pain, interventional procedures have to be taken.

The aim of this study was to evaluate the efficacy of using pulsed radiofrequency (PRF) on the dorsal root ganglia at the L4 and L5 nerve roots to improve phantom pain.

Two patients with phantom pain were selected for the study. After a positive response to segmental nerve blockade at the L4 and L5 nerve roots, PRF was performed on the L4 and L5 dorsal root ganglia.

Global clinical improvement was good in one patient, with a 40% decrease in pain on the visual analogue scale (VAS) in 6 months, and moderate in the second patient, with a 30% decrease in pain scores in 4 months. PRF of the dorsal root ganglia at the L4 and L5 nerve roots may be an effective therapeutic option for patients with refractory phantom pain.

## 1. Introduction

Phantom limb pain is a perception of a painful sensation that feels as if it originated from the missing limb after amputation. Phantom limb pain can appear in three possible ways:

1. Phantom pain: painful sensation in the amputated limb
2. Phantom sensation: any sensation, other than pain, in the amputated limb
3. Stump pain: localized pain in the amputated region (1).

The location and characteristics of the sensation are important in controlling the pain. Stump pain usually coincides with the development of phantom limb pain (2). Phantom pain has intermittent quality, which demonstrates neuropathic features, such as gripping, burning, squeezing, and drilling, and can be more intense in the furtherest part of the phantom, such as fingers, hands, and toes (3). The incidence of phantom limb pain can be as high as 60 to 80% (1). In a previous study, the visual analogue scale (VAS) of patients ranged from 2 to 7 on a 10-point scale (4). At present, no proven diagnostic test exists to evaluate phantom pain or stump pain except for the physical abnormalities in physical examination (5, 6).

Conservative therapy consists of drug treatments such as nonsteroidal anti-inflammatory drugs (NSAIDs) and opioids. When conservative treatment fails, lumbar sympathetic blockade, pulsed radiofrequency (PRF) ablation of the stump neuronal or the spinal ganglion, or spinal cord stimulation could be considered (7). Operations such as rhizotomy, cordotomy, stump revision, and dorsal column stimulation have been unsuccessful in treating these conditions. Dorsal root entry zone (DREZ) lesioning for postamputation pain has a limited but definite place in the treatment of postamputation pain (8).

The phenomenon of phantom limb pain remains poorly understood. As of this writing, no successful treatment exists and these patients continue to suffer from significant pain and disabilities. Careful and systematic application of available treatment methods will help to diminish pain without causing significant morbidity related to poor treatments. Ongoing laboratory and clinical research will continue to improve our understanding of this complex type of pain and improve treatment in the future.

The aim of this study was to evaluate the efficacy of using pulsed radiofrequency on the dorsal root ganglia at the L4 and L5 nerve roots to improve phantom pain.

## 2. Case Presentation

In our case study we identified two patients with phantom pain. Both patients had phantom pain for more than 3 months and were not responding to conservative treatments. They had no history of drug abuse. Magnetic resonance imaging (MRI) of the lumbosacral was normal and they had no lumbar radiculopathy or facetogenic pain. Both patients had a VAS of more than 5 and reported that their pain began from the stump and radiated to the lumbar and then the leg. Neither of the patients used prosthesis. Their physical examinations were normal except for hyperalgesia in the stump location in one of the patients and allodynia in the other. In both cases the major nerve responsible for the phantom pain was the sciatic nerve. At first a diagnostic selective nerve root (L4 and L5) blockade was performed in aseptic condition. For mild sedation, 1 mg of midazolam and 50 µg fentanyl were administered intravenously. 

**Figure 1.**
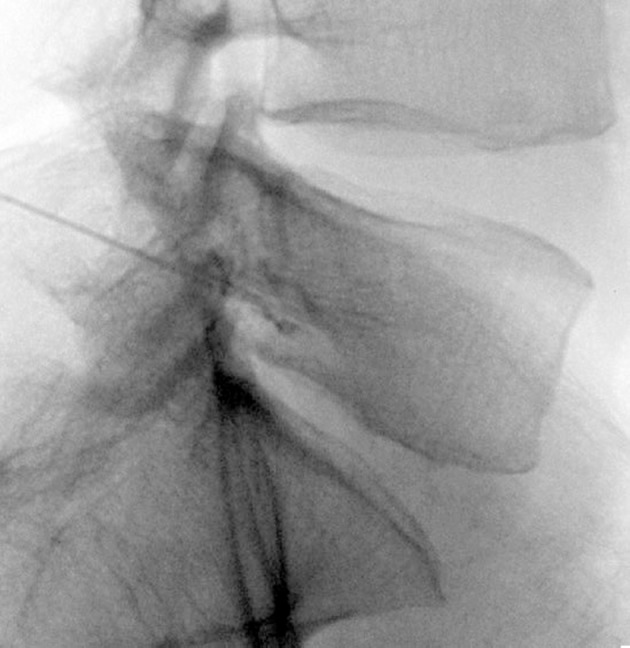
Radiofrequency Needle in Target Point

At first in the anterior- posterior (AP) view, the position of fluoroscope was changed to the cephalo-caudal direction to flatten the vertebrae end plate. Then, the C-arm was moved to an oblique position at a 20-degree angle. The target point in oblique view was below the pedicle at 6 o’clock. Local anesthesia was made by lidocaine (0.1%, 5 mL). Then, an introducer (Gauge 16) was inserted in tunnel view, its stylet was removed, and a blunt, curved-tip needle (Gauge 22 and 150 mm in length) was placed through the introducer. After confirming the tip of the needle was inserted in the oblique, AP, and lateral views (in the lateral view, in conjunction with the upper one-third and lower two-thirds in the upper lateral quadrant of the intervertebrae foramen), non-ionic water soluble contrast media (omnipaque 320) was administered. After confirming that the needle was not into the vascular bed, ropivacaine (0.2%, 3 mL) and triamcinolone (40 mg) were injected under live fluoroscopy. Two weeks after the first diagnostic blockade which was administered to decrease the patients’ pain, a PRF was performed on the patients in the same way as the diagnostic blockade technique, except that a blunt, curved-tip radiofrequency needle (Gauge 22, 150 mm in length, with a 10-mm active tip) was inserted. After confirming the position of the tip of the needle (dorso-cranial quadrant of the intervertebral foramen), a radiofrequency probe was placed instead of the stylet needle. Sensory stimulation was made (50 Hz, 0.4-0.6 voltage, 1-ms pulse width), which resulted in paresthesia in the stump or the amputated leg (sensation in the leg and fingers). Because the PRF did not have any significant side effects for the motor nerves, and it was impossible to achieve motor response for the amputated extremity, the functional test was not performed. A PRF was conducted on the dorsal root ganglia (DRG) in two cycles, 42 °C, 120 seconds in both L4 and L5. Then ropivacaine (0.2%, 3 mL) and triamcinolone (40 mg) were injected. This technique was performed for L4 and L5 in one patient but only for L4 in the other patient due to disturbed vertebral anatomy caused by hemipelvectomy (Figure 1). 

### 2.1. Case Report 1

A 35-year-old woman had an amputation right above the knee 3 months ago because of osteosarcoma. The chief complaint was intermittent and sharp right-side stump pain with radiation to the lumbar and then the right leg. She had phantom pain and phantom sensations. In the physical examination her stump had only a little hyperalgesia at one point. Also, the pain sometimes radiated to the left posterior thigh. Her lumbosacral MRI was normal. After diagnostic blockade, her stump pain completely disappeared, but the phantom sensation did not show any changes, and the patient reported that, after the blockade, she felt sensation again in what would have been the toe area of her right amputated leg. At first, she was very satisfied with the blockade, and a PRF of the right dorsal root ganglion was performed on this patient at L4 and L5. She reported a 40% decrease in pain on the VAS within 6 months.

### 2.2. Case Report 2

A 25-year-old woman presented with a history of left hemipelvectomy due to an accident from her childhood. Her chief complaint was left-side stump pain and right-side phantom pain. She had intermittent electrical-shock-like sensations shooting from her left stump up to her lumbar and then to her right leg. The pain sensations had gradually increased from once a year to every day. Her pain score (VAS) was about a 5 or 6 out of 10. Her physical examination was normal except for allodynia in the stump (at one point). Her lumbosacral MRI was normal. After diagnostic selective nerve root (L4) blockade, she underwent a PRF of the left L4 dorsal root ganglion (L5 was not possible due to anatomic distortion). She was satisfied and reported a 30% decrease in her VAS in 4 months.

## 3. Conclusions

The PRF on the L4 and L5 dorsal root ganglia was somehow effective in controlling phantom pain in the patients.

Dorsal root ganglia transfer sensory signals to the central nervous system, and they play a very important role in the pathogenesis of chronic pain syndrome. The application of PRF by creating an electromagnetic field has found a very special place in controlling chronic pain. This method can be used on lumbar dorsal root ganglia in controlling lumbosacral and stump pain (9-11). Dorsal root ganglia show abnormal spontaneous activity and increased sensitivity to mechanical and chemical stimulations. Their sodium channels tend to go through some changes, which in turn change the messages that are sent from the brain and peripheral area to the dorsal roots. These changes include an increase in sodium channels, a decrease in potassium channels, and activation of the calcium channels. Consequently, pain receptors become more sensitive, the field of sensation widens, central inhibition decreases, and the spinal cord is stimulated. Changes would also occur in the dorsal horn, and all of these effects will result in the experience of pain (10, 12, 13). All of these changes transform in a PRF electrical field, and pain thereby decreases. Controlling phantom pain is very difficult because we still do not know all of the mechanisms through which it is produced. However, some mechanisms have been identified, such as the effective mechanism on peripheral elements of the spinal cord, dorsal ganglia, and brain. Neuropathic changes of these segments are effective in controlling phantom pain. It seems that, at first, the peripheral nervous system is affected, then the central nervous system, and then the brain, and this order complicates the pain (1). Following amputations, some changes occur in the dorsal root, which experiences disinhibition, the depletion of Substance P, and a change in endorphins, all of which cause pain in the amputated limb (5).

Electric field by PRF causes different effects on the tissues. This electric field penetrates into the axon’s membrane and dorsal root ganglia and raptures important subcellular structures that will change or disable cell activities. This field changes the activity and behavior of the Schawnn cells and causes neural excitation. Also, by effecting the ions’ oscillations and polarization of the cells’ membranes, the field modifies cell structure and decreases pain messages by long-term depression of the dorsal root synaptic connections, which results in analgesia (14). A PRF on dorsal root ganglia causes cell activation in Layers 1 and 2 of the dorsal root and results in analgesia (15, 16). Also this analgesia is marked by an increase of ATF3 in small nerves (17). So, therefore, a PRF on dorsal root ganglia will result in analgesia and a decrease in neuropathic pains such as stump and phantom pain. A PRF on dorsal root ganglia is noninvasive, with no sensation of pain or functional complications and is an effective method in treating chronic pain.

A PRF on dorsal root ganglia is effective in controlling pain after thoracic surgery and stump pain of amputated limbs (9, 18). For this reason, we use PRF in controlling phantom pain. Finally, because phantom and stump pain are usually experienced together, controlling stump pain is effective in controlling phantom pain (2, 19, 20).
